# Predictors of insomnia among undergraduate students at Hawassa University Sidama, Ethiopia, 2023: a facility-based cross-sectional study

**DOI:** 10.3389/fpsyt.2024.1352291

**Published:** 2024-08-15

**Authors:** Mastewal Aschale Wale, Yared Reta, Haymanot Addis, Rahel Tarekegn, Mintesnot Tafese, Aklile Tsega Chekol

**Affiliations:** Faculty of Health Sciences, College of Medicine and Health Sciences, Hawassa University, Hawassa, Ethiopia

**Keywords:** sleep problem, prevalence, social media, Hawassa, undergraduate students

## Abstract

**Background:**

Insomnia is a sleep disorder characterized by difficulty falling asleep, staying asleep, or experiencing poor-quality sleep. People with this problem often have trouble falling asleep at night, wake up frequently during the night, and may wake up too early in the morning and feel tired and not refreshed. This can lead to daytime fatigue, irritability, difficulty concentrating, and impaired functioning in their day-to-day activities. Study is scarce in resource-limited countries such as Ethiopia, particularly concerning the study setting. As a result, this study aimed to assess the prevalence of insomnia and its associated factors among undergraduate students at Hawassa University.

**Methods:**

An institution-based cross-sectional study design was used. A stratified simple random sampling method was used among 398 study participants. The data were collected using a structured self-administered questionnaire. The outcome variable was assessed by the insomnia severity index (ISI). The data were then gathered by using the Kobo toolbox online and then exported into the Statistical Package for Social Sciences version 27 to analyze the data. Data cleaning and screening were conducted exclusively by the investigators. Descriptive statistics like frequency, percentages, and mean were used. Bivariate and multivariate binary regression were performed. In multivariate binary logistic regression, a *p*-value of<0.05 was identified as a significantly associated factor with the dependent variable.

**Results:**

Among 398 students who participated in the study, 81 (20.4%) experienced insomnia. Being female [adjusted odds ratio (AOR) = 2.98; 95% confidence interval (CI) 1.56–5.69], age (AOR = 3.06; 95% CI 1.11–8.45), mild anxiety symptom (AOR = 4.89; 95% CI 1.56–15.37), and mobile device use ≥30 min just before going to sleep (AOR = 7.81; 95% CI 2.34–26.12) were significantly associated with insomnia.

**Conclusion:**

In this study, the prevalence of insomnia was one-fifth among university students, which is high when compared to another study at the national level. There were significant associations between being female, age, anxiety symptoms, and mobile device use before going to sleep with insomnia. This indicates that there is a need to treat and prevent insomnia in college students, emphasizing the necessity for mental healthcare and ethical technology use.

## Background

Insomnia is defined as difficulty initiating or maintaining sleep that is associated with consequences of daytime activities and is not attributable to environmental circumstances or inadequate opportunity to sleep (1). It is characterized by chronic dissatisfaction with sleep quantity or quality that is associated with difficulty initiating and maintaining sleep, frequent nighttime awakenings with difficulty returning to sleep, not getting any restorative or reviving sleep, and awakening earlier in the morning than desired. In the general community, it is the most frequent sleep problem. It affects 33% to 50% of the adult population and 5% to 10% of the general population (2).

Sleep disturbances are a common complaint among college students worldwide, likely as a result of stress due to increased academic demands. Moreover, busy schedules, new social opportunities, and a sudden change in the sleeping environment can be additional contributing factors (3). The transition from high school to university presents many challenges, including leaving home, increased independence, changes in peer groups, new social situations, maintenance of academic responsibilities, and increased access to alcohol and drugs. Some students may cope more effectively with these stressors than others, and the latter may be at increased risk of developing insomnia. While recent research has helped to increase the public understanding of the importance of good sleep behaviors in young adults, a thorough investigation of insomnia and its correlation to university students is still lacking (4).

People with poor sleep quality are more likely to experience physical and psychosocial health problems, as well as lack of concentration, fatigue, irritability, anxiety, and depression (5). People with insomnia often report having difficulties with initiating and maintaining sleep, early morning awakenings, and sleep that is not refreshing. The high incidence of the condition is not helped by a prevailing attitude among many patients that insomnia is only one of the many challenges of life and is therefore not a “real” health problem that necessitates consultation with medical professionals (6).

Insomnia has notable consequences with regard to learning ability and academic success in higher education. It is associated with a higher risk of failed examinations and experiencing delayed study progress (5). Up to 60% of all college students suffer from poor sleep quality, and 7.7% meet all criteria of an insomnia disorder. Sleep problems have a great impact on the student’s daily life such as the student’s grades, regular daytime routines, chronotype changes, side jobs, and exam periods (7). Inadequate sleep leads to increased drowsiness and daytime sleepiness, which subsequently decreases mental alertness and concentration. This can affect the ability to deal with tasks involving problem-solving, memory, and attention to detail. Thus, students who suffer from sleep disorders are at a higher risk of failing academically, with lower grade point averages (GPAs) of<2.0 (8).

On the one hand, physiological factors, schooling, and work schedules affect sleep quality. On the other hand, poor sleep quality affects human cognitive functions, such as information processing, learning, and the integration of intellectual records. Poor sleep quality is highly correlated with poor academic performance and reduced learning ability to perform basic activities, such as solving a mathematical problem. Other detrimental effects of poor sleep quality include reduced memory, reduced cognitive ability, risk of suicide, mental problems, and poor sleep hygiene practices (3).

The prevalence of insomnia in various countries was found to be 40.8% among medical students in Pakistan (9), 12.1% at universities in the Netherlands (10), 19.3% at Jazan University (11), 32.5% at universities in Nigeria (12), and 61.6% at Debre Berhan University (3).

The purpose of this study is to assess the prevalence of insomnia and its associated factors among undergraduate students at Hawassa University by answering the following questions: What is the prevalence of insomnia in university students? Is there an association between the outcome variable and explanatory variables?

## Materials and methods

### Study area, period, and design

An institution-based cross-sectional study was conducted from July to August 2023 at Hawassa University located in Hawassa City, Sidama region, Ethiopia. The city is 285 km away from Addis Ababa, the capital city of the country. Hawassa University has seven campuses, namely, the main campus, the Institute of Technology, the College of Agriculture, the College of Medicine and Health Sciences, the Daye Campus, the Wondo Genet College of Forestry and Natural Resources, and the Awada Campus. Data were collected from three randomly selected campuses: the main campus, the College of Agriculture, and the College of Medicine and Health Sciences.

### Study population

All undergraduate students currently enrolled at Hawassa University were the source population. All randomly selected undergraduate students during the data collection period were the study population.

### Inclusion and exclusion criteria

All regular undergraduate students who enrolled in the second semester at Hawassa University at the selected campuses and who are available during the data collection period were included in the study, while students who are on an annual break, practice, and students who have no smartphones to complete the questionnaire were excluded from the study.

### Sample size determination and procedure

The sample size was determined by using a single proportional formula under the following assumptions: a proportion of 61.6% from a previous study in Ethiopia at Debre Berhan University (3) with a 5% margin of error at 95% confidence interval (CI).


$$n=\frac{{\left(za/2\right)}^{2}p(1-p)}{d^{2}},n=\frac{{\left(1.96\right)}^{2}[0.62(1-0.62)}{{\left(0.05\right)}^{2}}=362$$

The final sample size was 398 after adding a 10% non-response rate.

A stratified sampling technique was employed. First, three colleges were chosen using a simple random sampling technique using 40%. The registrar of each college provided an updated sampling frame of students in each department. The framework contained student names, sexes, departments, and student IDs. According to the information obtained from the registrar’s office, there were a total of 27,146 students on three campuses (23,062 students were from the main campus, 2,714 students were from the College of Medicine and Health Sciences, and 1,370 students were from the College of Agriculture). Proportional allocation was done for the three campuses (the main campus, the College of Medicine and Health Sciences, and the College of Agriculture) using a simple random sampling method based on the sample size. Then, we selected a proportional number of students from each stratum, in which we first made several lists of all units (sampling frame); a total of 27,146 students (23,062 from the main campus, 2,714 from the referral campus, and 1,370 from the agriculture campus) and 398 participants will be selected by using the lottery method (338 students from the main campus, 40 students from the referral campus, and 20 students from the agriculture campus by proportional allocation).

### Data collection tool

The data were collected by three BSc Psychiatry nursing professionals using a semi-structured self-administered questionnaire through the Kobo toolbox. The questionnaire has five sections. The first part included the socio-demographic characteristics of the study participants. The second part is the insomnia severity index, which is a brief screening assessment tool designed to evaluate insomnia. The insomnia severity index is a seven-item self-report tool used to evaluate the type, severity, and effects of insomnia (13, 14). The dimensions assessed the severity of sleep onset, maintenance, early morning awakening problems, sleep dissatisfaction, and interference of sleep difficulties with the seven items. The ISI is a self-report tool used to evaluate the type, severity, and effects of insomnia. Each question is rated on a five-point Likert scale (0 = no difficulty; 4 = extremely severe problem). Items are added together to get the final score, which can range from 0 to 28. The interpretation of the total score is as follows: 0–7 indicates no insomnia; 8–14 indicates sub-threshold insomnia; 15–21 indicates moderate insomnia; and 22–28 indicates severe insomnia (13, 15). This survey can be used for both screening and evaluating the effectiveness of treatments in clinical trials (13). Higher scores correspond to a more severe feeling of sleeplessness. It is valid in the Ethiopian adult population with moderate to significant item-total ISI score correlations (*r* = 0.47), as well as internal homogeneity and consistency (Cronbach’s alpha = 0.68 and 0.78) (16).

The third section contains the Hospital Anxiety and Depression Scale (HADS). It is a 14-item scale with seven items for each anxiety and depression subscale. HADS is validated and used in Ethiopia (17, 18). Scoring for each item ranges from 0 to 21. A subscale >8 denotes anxiety or depression (19, 20). Mobile-related sleep risk factors (MRSRF) are questionnaire items that focus on the total duration of mobile use per day, using a mobile device while in bed when the lights have been turned off, using blue light filters on mobile phones, keeping the mobile device under the pillow, keeping the mobile device 2 m away from the bed, and putting the mobile device on airplane mode while sleeping (21). These have been used in previous studies (22–25). The fourth section contains the Alcohol, Smoking, and Substance Involvement Screening Test (ASSIST), which was developed under the auspices of the World Health Organization (WHO) by an international group of addiction researchers and clinicians in response to the overwhelming public health burden associated with psychoactive substance use worldwide. It is an eight-item questionnaire and a risk score is determined for each substance by discussion with clients about their substance use. The score obtained for each substance falls into a “lower”, “moderate”, or “high” risk category, which determines the most appropriate intervention for that level of use (“no treatment”, “brief intervention”, or “referral to specialist assessment and treatment”, respectively) (26). It is utilized in Ethiopia (27).

### Data quality measure

To ensure the quality of the data, special attention was taken by ensuring that the students clearly understood the instructions about answering the questionnaire and written informed consent was given to the study participants. The participants were also informed that they would not be forced to do anything against their choice and that their information was kept completely secret. Moreover, before and throughout data processing, the information was checked for completeness, accuracy, and clarity as well as for correct collection and recording. The questionnaire was pre-tested among 5% of graduate students at the IOT campus 2 weeks before the actual data collection period. The internal consistency (Cronbach’s alpha) of the tool in this study was 0.886.

### Data processing and analysis

The data were checked for completeness and consistency and then coded. The coded data were loaded into the Statistical Package for Social Sciences version 25 and analyzed using it. Bivariate and multivariate binary regression were performed. In binary logistic regression, variables with *p*-value< 0.25 were candidates for multivariate logistic regression. Statistical significance was declared at a 95% CI when variables have a *p*-value< 0.05 in the multivariate analysis with premenstrual dysphoric disorder. Finally, a compiled result was presented in the form of text, tables, and graphs of the characteristics of the study subjects.

### Operational definition

Insomnia: Students who scored >15 on the insomnia severity index have insomnia (28).

Common mental illness: using HADS, students who scored ≥8 have depression and anxiety (19).

Substance use: using ASSIST, students who scored 0–3 (0–4 for cannabis) need brief education, those who scored 4–26 (5–26 for cannabis) need brief intervention, and those who scored 27+ need brief intervention offer options that include treatment (29).

## Results

### Socio-demographic characteristics of respondents

From the total number of 398 distributed questionnaires for study participants, all were filled out completely and consistently with a response rate of 100%. Out of this, more than half of the participants (216, 54.3%) were men. The minimum and maximum age of the participants was 18 and 28, respectively, with a mean age of 22.22. More than one-third of the participants (158, 39.7%) were orthodox religious followers. Nearly three-fourths (283, 71.1%) of the participants were single (**Table 1**).

**Table 1:** Socio-demographic characteristics of undergraduate students at Hawassa University Hawassa, Southern Ethiopia, 2023 (*n* = 398).

Variables	Categories	Frequency	Percentage (%)
Sex	Male	216	54.3
	Female	182	45.7
Age	18–22	320	80.4
	23–28	78	19.6
Religion	Orthodox	158	39.7
	Muslim	68	17.1
	Protestant	122	30.7
	Catholic	41	10.3
	Other*	9	2.3
Student marital status	Single	283	71.1
	In a relationship	106	26.6
	Married	9	2.3
Parental marital status	Living together	246	61.8
	Divorced	74	18.6
	Widowed	61	15.3
	Both lost in death	17	4.3
Residency	Dormitory	241	60.6
	Home	125	31.4
	Rent house	32	8
Field of study	Natural and computational science	188	47.2
	Law	81	20.3
	Other health science	25	6.2
	Agricultural science	20	5.02
	Medicine	15	3.5
	Other**	69	17.58

^*^Jehovah’s witness and atheist; ^**^Journalism, hotel management, special needs, and sociology.

### Common mental illness-related factors

From the 398 participants, more than two-thirds (271, 68.1%) had depression symptoms and 281 (70.6%) of them had anxiety symptoms (**Table 2**).

**Table 2:** Hospital anxiety and depression scale among undergraduate students at Hawassa University, Hawassa, Southern Ethiopia, 2023 (*n* = 398).

Depression	Yes Definitely	Yes Sometimes	No, not much	No, not at all
I wake up early and then sleep badly for the rest of the night.	36 (6%)	166 (41.7%)	142 (35.7%)	54 (13.6%)
I feel miserable and sad.	12 (3%)	159 (39.9%)	147 (36.9%)	80 (20.1%)
I have lost interest in things.	21 (5.3)	143 (35.9%)	153 (38,4%)	81 (20.4%)
I have a good appetite.	142 (35.7%)	106 (26.6%)	127 (31.9%)	23 (5.8%)
I feel life is not worth living.	15 (3.8%)	129 (32.4%)	156 (39.2)	98 (24.6%)
I still enjoy the things I used to.	100 (25.1)	159 (39.9%)	109 (27.4%)	30 (7.5%)
I feel as if I have slowed down.	16 (4%)	135 (33.9%)	156 (39.2%)	91 (22.9%)
Anxiety				
I get very frightened or have panic feelings for apparently no reason at all.	26 (6.5%)	154 (38.7%)	140 (35.2%)	78 (19.6%)
I feel anxious when I go out of the house on my own.	31 (7.8%)	147 (36.9%)	141 (35.4%)	79 (19.8%)
I get palpitations, or sensations of “butterflies” in my stomach or chest.	20 (5%)	127 (31.9%)	146 (36.7%)	105 (26.4%)
I feel scared or frightened.	21 (5.3%)	142 (35.7%)	142 (35.7%)	93 (23.4%)
I am restless and cannot keep still.	19 (4.8%)	113 (28.4%)	160 (40.2%)	106 (26.6%)
I am more irritable than usual.	20 (5%)	158 (39.7%)	147 (36.9%)	73 (18.3%)
Worrying thoughts constantly go through my mind.	63 (15.8%)	184 (46.2%)	99 (24.9%)	52 (13.1%)

### Social media-related factors

More than three-fourths of the participants (356, 89.4%) use their mobile device just before going to sleep, and from these participants, 198 (49.7%) use their mobile device for ≥30 min just before going to sleep. More than three-fourths of the participants (355, 89.2%) keep their mobile device on their bed near their pillow. Nearly two-thirds of the participants (259, 65.1%) use a blue light filter or night mode on their mobile device. Three-fourths of the participants (305, 76.6%) do not put their mobile device on airplane mode while sleeping (**Table 3**).

**Table 3:** Social media-related respondents in Hawassa University, Hawassa, Southern Ethiopia, 2023 (n = 398).

Variable	Categories	Frequency	Percentage (%)
For how many total hours/24 h do you use a mobile screen?	<8 h	183	46%
	≥8 h	215	54%
How many hours do you spend watching videos on YouTube?	<3 h	188	47.2%
	≥3 h	210	52.8%
Do you use your mobile just before going to sleep (while in bed, when the lights have been turned off)?	Yes	356	89.4%
	No	42	10.6%
If yes to the above question, for how many hours do you use	0	44	11.1%
	<30 min	156	39.2%
	≥30 min	198	49.7%
Do you keep your mobile on your bed (near your pillow) while sleeping?	Yes	355	89.2%
	No	43	10.8%
Do you keep your mobile away from your bed (at least 2 m away) while sleeping?	Yes	40	10.1%
	No	358	89.9%
Do you put your mobile on airplane mode while sleeping?	Yes	93	23.4%
	No	305	76.6%
Do you use blue light filters (night mode) on your mobile?	Yes	259	65.1%
	No	139	34.9%

### Substance-related factors

Of the total participants, more than half (231, 58%) never used any substance in their lifetime while more than one-fifth (89, 22.4%) of the participants used alcohol (**Table 4**).

**Table 4:** Substance-related questions of respondents in Hawassa University, Hawassa, Southern Ethiopia, 2023 (n = 398).

In your life, which of the following substances have you ever used?	Response	Percentage
Tobacco products (cigarettes, chewing tobacco, cigars, etc.)	28	7%
Alcoholic beverages (beer, wine, spirits, etc.)	89	22.4%
Cannabis (marijuana, pot, grass, hash, etc.)	5	1.3%
Amphetamine-type stimulants (speed, meth, ecstasy, etc.)	30	7.5%
Inhalants (nitrous, glue, petrol, paint thinner, etc.)		
Sedatives or sleeping pills (diazepam, alprazolam, flunitrazepam, midazolam, etc.)	2	0.8%
Hallucinogens (LSD, acid, mushrooms, trips, ketamine, etc.)	1	0.3%
Opioids (heroin, morphine, methadone, buprenorphine, codeine, etc.)	9	2.3%
Any other drugs	3	0.8%
Never used any of the above substance	231	58%

### Prevalence of insomnia

**Figure 1:**
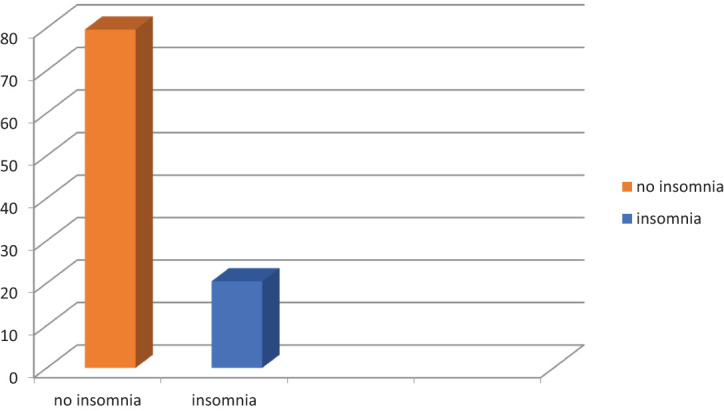
Prevalence of insomnia among Hawassa University undergraduate students, Hawassa, Southern Ethiopia, 2023 (n = 398).

Among 398 students who participated in the study, 20.4% [95% CI 16.3–24.1] experienced insomnia. Psychoeducation about good sleep hygiene was given to those students experiencing such a problem (**Figure 1**).

### Factors associated with insomnia

To investigate the association of independent variables with insomnia, both bivariate and multivariate binary analyses were used. In the bivariate binary logistic regression analysis, sex, age, parental marital status, depression, anxiety, use of mobile phone just before going to sleep when the lights have been turned off, duration of mobile phone use just before going to sleep, and using blue light filters or night mode were the candidates for multivariate logistic regression to adjust the possible confounders with a *p*-value of ≤0.25. However, sex, age, anxiety, and duration of mobile phone use just before going to sleep had a *p*-value of<0.05 in multivariate binary logistic regression and were found to be statistically significant.

Female students were three times more likely to develop insomnia than male students [adjusted odds ratio (AOR) = 3.09; 95% CI 1.62–5.89]. Students who belong to the 18–22 age group are approximately two times more likely to develop insomnia as compared to those who are in the older age group (AOR = 2.93; 95% CI 1.08–7.93). Students who had anxiety symptoms were 10 times more likely to develop insomnia as compared to students who had no anxiety (AOR = 10.60; 95% CI 2.84–39.56). Students who use their mobile device for ≥30 min just before going to sleep when the lights have been turned off are six times (AOR = 6.085; 95% CI 1.077–34.73) more likely to develop insomnia as compared to students who do not use their mobile device just before going to sleep when the lights have been turned off (**Table 5**).

**Table 5:** Bivariate and multivariate binary logistic regression analysis of factors associated with insomnia among undergraduate students at Hawassa University, Hawassa, Southern Ethiopia, 2023 (n = 398).

Variable	Category	Insomnia		COR (95% CI)	AOR (95% CI)	p-value
		Yes	No			
Sex	Female	52	130	2.58 (1.55–4.28)	**3.09 (1.62–5.89)**	<0.001**
	Male	29	187	1		
Age	18–22	75	245	3.67 (1.54–8.79)	**2.93 (1.08–7.93)**	0.034**
	23–28	6	72	1		
Parental marital status	Living together	1	9	1		
	Divorced	22	56	3.54 (0.423–29.58)	1.143 (0.11–11.922)	0.911
	Widowed	39	210	1.67 (0.21–13.57)	0.56 (0.56–5.64)	0.626
	Both lost in death	19	42	4.07 (0.48–34.46)	2.33 (0.22–24.72)	0.481
Anxiety	No	114	3	1		
	Yes	203	78	14.60 (4.51–47.32)	**10.60 (2.84–39.56)**	<0.001**
Depression	No	117	10	1		
	Yes	200	71	4.16 (2.06–8.37)	2.46 (0.99–6.14)	0.053
Using a mobile device just before going to sleep when the lights are turned off	Yes	78	278	3.65 (1.10–12.12)	0.69 (0.18–3.98)	0.64
	No	3	39	1		
Duration of mobile device use just before going to sleep	0	3	41	1		
	<30 min	6	150	0.55 (0.13–2.28)	0.26 (0.04–1.70)	0.159
	≥30 min	72	126	7.81 (2.34–26.12)	**6.09 (1.08–34.37)**	0.041**
Using blue light filters or night mode	Yes	58	201	1		
	No	23	116	1.46 (0.85–2.48)	0.0.75 (0.37–1.53)	0.423

AOR, adjusted odds ratio; COR, crude odds ratio; CI, confidence interval; 1, reference group; *p-value< 0.05.

The single asterisk shows p-value less than 0.25 and the double asterisk shows p-value less than 0.05.

## Discussion

Good emotional and social functioning, as well as one’s physical and mental health, depend on getting enough sleep, and insomnia can have several negative effects. One of the most prevalent sleep disorders, insomnia can hurt a person’s emotional and general wellbeing. It is also linked to several health issues, including a higher risk of obesity, diabetes, heart disease, and stroke. The definition of insomnia, the screening instrument used to assess it, and the population under study all affect how common insomnia is. A clinical syndrome of insomnia is present in 5%–10% of individuals, and 30%–50% of individuals have one or more of the symptoms needed to diagnose insomnia (30). The prevalence of insomnia among regular undergraduate students at Hawassa was 20.4%. In the multivariate logistic regression analysis, being female, younger age, having anxiety symptoms, and using a mobile phone device for more than 30 min were statically significant factors associated with insomnia.

The results of this study indicate that 20.4% (95% CI 16.3–24.4) of respondents had insomnia. This study is consistent with a previous study conducted at Jazan University, Southwestern Saudi Arabia (where 19.3% of respondents had insomnia) (11). Both studies utilize similar tools to assess insomnia. In this study, the prevalence of insomnia was higher compared to a previous finding reported in the University of North Texas (9.5%) (5), Cumhuriyet University in Turkey (12.1%) (31), Iran (9.79%) (32), and USA (12%) (33). The difference might have occurred due to the difference in methods used, such as sample size and assessment tools. On the other hand, the prevalence of the current study was lower than that of studies involving Debre Berhan University (61.1%) (3), Pakistan Sheikh Zayed Medical College and Hospital (40.8%) (9), Omani University (79.3%) (34), Jordan University (60.6%) (35), Malaysian students (69%) (36), and Norway University (30.5%) (37). The difference might have occurred due to the different measurement tools used to assess insomnia in studies. The possible rationale could be several variables contributing to insomnia, involving psychological, social, cultural, and physical aspects (38–40).

Students who were younger than 25 years old had a higher likelihood of experiencing sleeplessness compared to those who were older than 25 (AOR = 2.93; 95% CI 1.08-7.93). This is supported by studies conducted at Mizan Tepi University (6), Norway (37), and Saudi Arabia (41). Most young individuals experience insomnia while attending college, likely as a result of the elevated stress levels that are typical of college students (42, 43). Additionally, another possible reason might be some circumstances may have contributed to the development of these illnesses. For instance, younger students who are experiencing college life for the first time must adjust to some significant changes in their sleeping environment, an unfamiliar type of housing, the anxiety of being away from home, a shift to higher performance standards in the classroom, and pressure from peers and family to perform well academically. All of these elements have the potential to cause people to experience ongoing stress, which can lead to complaints linked to insomnia and ongoing sleep loss. The other possible rationale could be comparable patho-psychophysiological processes at work, the effects of which are visible despite socioeconomic differences. Another explanation could be that there are possibly contributing factors to the issue among younger pupils. For example, first-year university students have to adjust to several big changes, like sudden changes in their sleeping schedules, a new type of housing, the anxiety of being away from home, a move to a higher level of academic performance, and pressure from family and friends to do well in their studies (44). Regular screenings for insomnia and psychoeducation regarding good sleep hygiene habits for college students should be conducted by the university health centers. More significantly, tailored approaches to managing insomnia in this population may be aided by the modifiable risk factors of insomnia, such as good sleep hygiene.

On the other hand, the current study was contradicted by the study conducted in China (45). Students who belong to an older age group (>25) are more likely to develop insomnia than those belonging to a younger age group (45). The possible explanation may be due to differences in the personality of students who find it easy to adapt to new learning areas easily, the lifestyle of the students, and differences in geographical areas.

In this study, female students are more likely to develop insomnia than male students (AOR = 3.09; 95% CI 1.62–5.89). This is consistent with studies done in (8, 9, 41, 46–49). The possible explanation for this finding is that women tend to be more affected by sleep complaints compared to men; this has been attributed to the increased frequency of other conditions affecting sleep, such as stress and anxiety, among women. There are gender disparities in insomnia and sex-related psychological health, and women are reported to experience anxiety at a higher rate (50–52). Men regarded work-related causes as the most significant cause of sleep disturbance, whereas women perceived psychological aspects as the most significant cause, according to another study on self-evaluations of factors disrupting sleep. Another finding of the current study was that those students with anxiety symptoms are more likely to have insomnia when compared to students without the symptom (AOR = 10.60; 95% CI 2.84–39.56). This is in line with some studies (34, 53). The possible justification might be when someone is anxious, their mind races with unfavorable thoughts and anxieties. In addition, since hypervigilance is the primary symptom of anxiety, people who are anxious find it difficult to relax and go to sleep. Physical signs of anxiety include sweating, tense muscles, and a racing heartbeat (5).

Students who spent more than 30 min of mobile device use just before going to sleep when the lights were turned off were significantly associated with the outcome variable (AOR = 6.09; 95% CI 1.08–34.37). It is consistent with a study conducted in Saudi Arabia (21). A possible explanation could be that students who were overburdened by their schoolwork stayed up late using their computers or cellphones, potentially disrupting their circadian cycle. Moreover, another rational explanation could be that university students use cellphones to read emails, access educational materials online, connect to various social media sites, and access papers pertinent to their studies (54). All things considered, the drawbacks of smartphone use include energy loss, sleeplessness, and the adoption of unhealthy habits (55).

## Limitations of the study

The study relies on self-reported data, which may be subject to recall bias or social desirability bias. The study does not explore other potential risk factors for insomnia, such as lifestyle factors or medical conditions.

## Conclusion

In this study, the prevalence of insomnia was one-fifth among university students, which is high when compared to another study at the national level. There were significant associations between being female, age, anxiety symptoms, and mobile device use before sleep with insomnia. To treat and prevent insomnia in college students, these findings emphasize the necessity for mental healthcare and ethical technology use.

## Recommendation

To reduce anxiety and sleeplessness, it may be more effective to employ student-centered counseling sessions that raise awareness and teach about managing sleep hygiene practices and other modifiable risk factors. This could potentially stop additional unfavorable secondary consequences. To improve students’ performance in their daily tasks, special attention should be paid to the following risky groups: female students, younger students, students who have anxiety symptoms, and students who use their mobile devices more than 30 min before bedtime after the lights have gone out.

## Data Availability

The raw data supporting the conclusions of this article will be made available by the authors, without undue reservation.
